# Supplementary material for: TongGuanWan Alleviates Doxorubicin- and Isoproterenol-Induced Cardiac Hypertrophy and Fibrosis by Modulating Apoptotic and Fibrotic Pathways
Source: Int J Mol Sci. 2024 Sep 30;25(19):10573. doi: 10.3390/ijms251910573 (PMC11476530; doi:10.3390/ijms251910573)
Supplement: Supplementary file 1 [file ijms-25-10573-s001.zip › ijms-3191017-supplementary.pdf]

Supplementary Data

TongGuanWan attenuates pathological Cardiac Hypertrophy via modulation of Fibrotic response

Jung Joo Yoon[1], Ai Lin Tai[1,2], Hye Yoom Kim[1], Byung Hyuk Han[1], Sa Rah Shin[3], Ho Sub Lee[1], Dae Gill Kang[1,2]

[1]Hanbang Cardio-Renal Syndrome Research Center, Wonkwang University, 460, Iksan-daero, Iksan, Jeonbuk 54538, Republic of Korea

[2]College of Oriental Medicine and Professional Graduate School of Oriental Medicine, Wonkwang University, 460, Iksan-daero, Iksan, Jeonbuk 54538, Republic of Korea

[3]KM Science Research Division, Korea Institute of Oriental Medicine, 1672, Yuseong-daero, Yuseong-gu, Daejeon 34054, Republic of Korea.

Correspondence to: Ho Sub Lee or Dae Gill Kang, Hanbang Cardio-Renal Syndrome Research Center, Wonkwang University, 460, Iksan-daero, Iksan, Jeonbuk 54538, Republic of Korea

E-mail: host@wku.ac.kr (H. S. Lee)

E-mail: dgkang@wku.ac.kr (D.G. Kang)

UPLC/QE Orbitrap MS analysis of Compounds in TGW

A 0.10g of TGW was prepared in 1.5 ml e-tube and added 750 µl of 70% methanol (MeOH) for solvent extraction. After centrifugation at 13,200 rpm for 10 minutes at 4°C, 500 ul of supernatant was collected in fresh 1.5 ml e-tube. Briefly, 100 ul aliquots of this solution were diluted with 70% acetonitrile (ACN) until 10ppm for ingredients analysis.

The UPLC/QE Orbitrap MS which was equipped with a heated electrospray ionization (HESI) source was used. Chromatographic separation were performed on a Acquity UPLC CSH™ C18 (2.1 x 100 mm, 1.7 um, Waters, USA) column, using binary gradient system. The mobile phase comprised 0.1% formic acid (FA) in distilled water (DW, solvent A) and ACN (solvent B) and elution gradient was follows; 20 to 60% B; 0-8 min→60 to 90% B; 8-10

min→90 to 98% B; 10-11 min and hold 1 min→98 to 20% B; 12-13 min→ equilibration until 20 min. Separation were accomplished at a column flow of 0.35 ml/min and temperature of 40°C. The injection volume of samples was 5 µl and autosampler were kept at 5°C. All analyzes were performed in both ionization modes (negative and positive) based on Full MS and data dependent MS2 (dd-MS2) scan mode (m/z 100-1200).

UPLC/QE Orbitrap MS data (.raw) were converted file format (.abf) for processing MS-Dial (http://prime.psc.riken.jp/Metabolomics_Software/MS-DIAL) to determine features, perform the alignment, and generate peak tables of m/z and the retention time for samples. Identification of TGW contents based on the retention time, m/z, and the MS fragment pattern, using in public databases including Metlin (metlin-scripps.edu), the Human Metabolome Database (www.hmdb.ca) and MassBank of North America (http://mona.fiehnlab.ucdavis.edu). In addition, 4-hydroxy cinnamic acid, catapol, coumarin, ferulic acid, mangiferin, morroniside, neomangiferin, palmatine, and protocatechuic acid were confirmed using authentic compounds.

Supplementary Table S1.

Compounds	Adduct	Rt (min)	Observed (m/z)	Fragmention	Theoretical (m/z)	Accuracy (△ppm)
4-hydroxy cinnamic acid	[M-H]-	2.51	163.0398	119.0492	163.0401	-1.84
Adenosine	[M+H]+	0.67	268.1044	136.0627	268.1040	1.49
Asparagine	[M-H]-	0.71	131.0456	114.0184	131.0462	-4.58
Berberine	[M]+	2.69	336.1238	321.0977	336.1230	2.38
Catalpol	[M-H]-	0.82	361.1155	97.0282	361.1140	4.15
CocamidopropylBetaine	[M+H]+	7.21	343.2964	129.3673	343.2955	2.62
Coumarin	[M+H]+	3.92	147.0445	103.0546	147.0441	2.72
Ferulic acid	[M-H]-	2.78	193.0508	134.0365	193.0506	1.036
Glucose	[M-H]-	0.73	179.0560	89.0257	179.0561	-0.56
Maltotriose	[M-H]-	0.69	503.1641	221.0660	503.1618	4.57
Mangiferin	[M+H]+	1.10	423.0937	273.0391	423.0922	3.54

Mannitol	[M-H]-	0.73	181.0717	163.0602	181.0718	-0.55
Morroniside	[M-H]-	0.78	405.1417	101.0232	405.1437	-4.93
Neomangiferin	[M-H]-	0.78	583.1332	331.0471	583.1305	4.63
Palmatine	[M+H]+	2.67	352.1551	337.1302	352.1545	1.70
Phenylalanine	[M+H]+	0.78	166.0869	120.0814	166.0863	3.61
Protocatechuic acid	[M-H]-	1.12	153.0188	109.0284	153.0193	-3.27
Pyroglutamic acid	[M-H]-	1.05	128.0347	82.0283	128.0353	-4.69
Quinic acid	[M-H]-	0.82	191.0563	85.0301	191.0561	1.05
Stachyose	[M-H]-	0.73	665.2178	89.0233	665.2146	4.81
Sucrose	[M-H]-	0.73	341.1104	179.0553	341.1089	4.39
Trigonelline	[M+H]+	0.71	138.0554	94.0656	138.0550	2.89
Tryptophan	[M+H]+	0.85	205.0978	188.0711	205.0972	2.92
